# The impact of heart failure on patients and caregivers: A qualitative study

**DOI:** 10.1371/journal.pone.0248240

**Published:** 2021-03-11

**Authors:** Colleen A. McHorney, Sonal G. Mansukhani, Milena Anatchkova, Natalie Taylor, Heidi S. Wirtz, Siddique Abbasi, Lynwood Battle, Nihar R. Desai, Gary Globe

**Affiliations:** 1 Patient Centered Research, Evidera, Bethesda, MD, United States of America; 2 Global Health Economics, Amgen, Thousand Oaks, CA, United States of America; 3 Patient Author from Cincinnati, Cincinnati, OH, United States of America; 4 Yale School of Medicine, New Haven, CT, United States of America; Ospedale del Cuore G Pasquinucci Fondazione Toscana Gabriele Monasterio di Massa, ITALY

## Abstract

**Background:**

Heart failure is rising in prevalence but relatively little is known about the experiences and journey of patients and their caregivers. The goal of this paper is to present the symptom and symptom impact experiences of patients with heart failure and their caregivers.

**Methods:**

This was a United States-based study wherein in-person focus groups were conducted. Groups were audio recorded, transcribed and a content-analysis approach was used to analyze the data.

**Results:**

Ninety participants (64 patients and 26 caregivers) were included in the study. Most patients were female (52.0%) with mean age 59.3 ± 8 years; 55.6% were New York Heart Association Class II. The most commonly reported symptoms were shortness of breath (81.3%), fatigue/tiredness (76.6%), swelling of legs and ankles (57.8%), and trouble sleeping (50.0%). Patients reported reductions in social/family interactions (67.2%), dietary changes (64.1%), and difficulty walking and climbing stairs (56.3%) as the most common adverse disease impacts. Mental-health sequelae were noted as depression and sadness (43.8%), fear of dying (32.8%), and anxiety (32.8%). Caregivers (mean age 55.5 ± 11.2 years and 52.0% female) discussed 33 daily heart failure impacts, with the top three being reductions in social/family interactions (50.0%); being stressed, worried, and fearful (46.2%); and having to monitor their “patience” level (42.3%).

**Conclusions:**

There are serious unmet needs in HF for both patients and caregivers. More research is needed to better characterize these needs and the impacts of HF along with the development and evaluation of disease management toolkits that can support patients and their caregivers.

## Introduction

Chronic heart failure (HF) results when the heart cannot pump enough blood to meet the body’s needs [1]. HF is considered a global epidemic—with an estimated 64 million individuals affected worldwide and likely many more caregivers impacted—which is anticipated to drive healthcare costs up to USD 70 billion by 2030. Annually, HF diagnoses lead to more than one million hospitalizations and approximately 300,000 deaths in the United States (US) [2, 3].

Chronic HF is characterized by slow progression of symptoms that include breathlessness with exertion, shortness of breath, fatigue, tiredness or weakness, difficulty breathing when lying down, sleep problems, and swollen legs or ankles. These symptoms—especially shortness of breath and fatigue—diminish the health-related quality of life (HRQoL) of both patients and their caregivers as many patients are unable to function independently in their day-to-day lives [4]. Depression is also a common psychological sequalae of HF [5–9]. Furthermore, the day-to-day unpredictability of HF (i.e., acute exacerbations and hospitalizations), medical management issues such as pill burden and side effects can cause distress and feelings of hopelessness and helplessness in both HF patients and their caregivers [10]. While there are successful HF therapies which have demonstrated reductions in mortality and morbidity, very few HF drugs have been indicated for improving HRQoL in HF patients in addition to physical function, or symptoms [11].

Several studies have documented the impact of HF which results in substantial caregiver burden experienced by loved ones [12–18]. In other words, living with HF is a “shared experience” for both patients and caregivers [14]. Caregivers have reported experiencing distress, depression, anxiety, social isolation, and health problems related to caring for a patient with HF [12, 14]. Research has also demonstrated that, across various cardiovascular diseases, caregiver physical or emotional strain is an independent risk factor for caregiver mortality [19]. The totality of physical and emotional impacts on the caregiver of HF patients suggests that caregiver burden and well-being are important outcomes to consider for chronic HF management.

HF management is complex and requires daily coordination of and adherence to multiple medications and a set of lifestyle changes related to dietary restrictions, fluid intake, exercise, and weight monitoring. Frequent healthcare appointments—across several different provider types—are often necessary for HF patients. Caregivers play an important role in day-to-day HF management [16, 20, 21], and existing evidence suggests that the HF treatment journey is challenging for patients and caregivers alike [10, 12, 15–17, 22, 23]. As the US population continues to age, HF management will have an increasing impact on the caregivers of HF patients.

The goal of this research is to explore and present the symptom and symptom-impact experiences of HF patients and their caregivers. Unlike some other chronic diseases (such as hypertension or dyslipidemia), day-to-day symptom impacts in HF are not an experience and journey just of the individual patient. Rather, caregivers experience their own burden, strain, distress, and hardships by the act of caregiving for HF patients.

While past research has characterized the burden of HF on patients and caregivers, it is not without limitations, which motivated the current research. First, some studies were published 11–13 years ago [10, 24]. Treatments for HF have advanced across that period, perhaps making these more dated studies less salient and informative in terms of the patient and caregiver journey. Second, some sample sizes have been quite small (fewer than 20 patient and/or caregiver participants [13, 17, 23, 24]), which can limit their generalizability. Third, other past studies were conducted at single sites within a given country [15, 17, 22, 24], which also contributes to generalizability issues. Fourth, two studies were literature reviews with articles dating up to 20 years ago [14, 18]. Fifth, of the past qualitative studies, all were one-on-one interviews. While one-on-one interviews and focus groups each have inherent limitations, focus groups can often lead to more discerning discoveries because group interactions can yield more meaningful findings than individual interviews [25]. With these limitations and considerations in mind, we embarked on qualitative discovery research with 64 HF patients and 24 caregivers of HF patients sampled in 2017 and 2018 in three US cities (in different geographical regions) with an aim to cross-validate past studies and contribute new insights.

## Methods

### Study design and sampling

This was an observational, cross-sectional, qualitative, focus group study in the US wherein 19 in-person focus groups were conducted with 64 patients with HF and 26 caregivers. The study independently recruited patients and caregivers. In this article, the term “caregiver” was used to define anyone who cares—unpaid—for a friend or a family member living with HF.

Patients included in the study had to be at least 45 years old at the time of screening and self-reported the following: a diagnosis of HF, at least New York Heart Association (NYHA) class II, and taking at least one HF prescription medication in the last six months. Caregivers were eligible if they were at least 21 years old, a relative of any HF patient (not necessarily participating in the focus groups) with at least NYHA class II status who is not participating in the study, and a self-report of spending at least eight hours a week caring for the HF patient. All participants were recruited using market-research vendors across four US locations: Beverly Hills, CA; Skokie, IL; Boston, MA; and Philadelphia, PA. A standardized recruitment script was used by the vendors to identify potential participants from their proprietary databases. The sample was selected to represent as diverse a participant mix as possible regarding gender, age, socioeconomic status, and ethnicity. Male and female sessions were conducted separately as were patient and caregiver sessions. All procedures and patient-facing materials were approved by Ethical & Independent Review Services institutional review board (IRB), which is an independent ethics committee, before study initiation. All participants were consented verbally and in writing. The identification number for this study is 17191.

The patient and caregiver focus groups were conducted using semi-structured discussion guides. The patient guide was carefully designed to elicit physical and emotional sequelae, self-management, medication adherence, and manifestations of worsening and improvement in overall health status to understand participants’ symptom experience, impacts on day-to-day functioning, and solutions and resources that could facilitate the HF journey. For the caregiver groups, the guide was designed to understand length of experience, roles and responsibilities, extent of educational resources and social support, and facilitators and barriers to caregiving. Both discussion guides were reviewed by two HF patient advocates who participated in the study to provide feedback on the focus group questions. Table 1 gives illustrative questions that were used in the focus group guides. All focus group sessions were audio- and video-recorded (with participants’ permission) and lasted approximately 90 minutes.

**Table 1:** Illustrative questions in the discussion guides.

Core Topics Discussed	Focus Group Guides
Cardinal Symptoms and Impacts, and Elicitation of Non-cardinal Symptoms and Impacts	Example question: How has having chronic heart failure transformed or changed or impacted your life (i.e., the “new normal”)?
Medication Adherence	Example question: How well does your current treatment regimen treat the most significant symptoms of your disease?
Self-management	Example question: Do you feel like you have all of the information you need about chronic heart failure, its treatment, and its prognosis?
Quality of Care	Example question: How would you redesign the care and treatment you received for your chronic heart failure to include all the things you find important?

Following written informed consent, focus groups were conducted by one of the researchers (CAM) who used probing and non-verbal techniques to elicit responses from participants following the semi-structured interview guide.

All participants also completed a self-reported sociodemographic and clinical questionnaire. Recognizing the wealth of literature around HF symptoms and impacts, two small (8.5x11-inch) posters were developed that included the cardinal HF symptoms and impacts (S1 Appendix). This was utilized to cue the participants and act as a steppingstone to other discussion topics.

## Analysis

### Qualitative data analysis

All audio recordings of the focus group discussions were professionally transcribed. Upon receipt of each transcript, one researcher (NT) performed quality checks. The goal of this step was to correct any transcription errors and remove any personal health information found within the transcripts. A content-analysis approach was used to analyze the focus group discussion data (based on notes, transcripts, and audio recordings). All analyses were performed using an analysis software program (ATLAS.ti version 7.5.2). A coding dictionary was developed prior to qualitatively analyzing the transcripts to capture the symptoms and impacts elicited in the sessions. The coding dictionary was revised in vivo to include codes for new concepts. Once all transcript coding was complete, all codes were thoroughly reviewed by a second team member to ensure it was performed in an accurate and consistent manner. The qualitative output included the text captured by the coding process for each code and the participant quotes organized by HF symptoms and impacts from the perspective of patients and caregivers interviewed.

### Quantitative data analysis

A DataFax database for all quantitative data was developed, tested, and validated prior to data entry. DataFax is a 21 Code of Federal Regulations (CFR) Part 11-compliant, direct fax-to-computer data management system that relies on optical character recognition (OCR) software for collecting study data from case report forms. The quantitative data entered by the OCR software was reviewed by two independent reviewers to ensure accuracy. Descriptive statistics (number, mean, standard deviation [SD], and frequency) were presented for the sociodemographic and clinical form items.

## Results

### Patients

A total of 64 patients who met the eligibility criteria participated in the one-time, focus-group discussion. Focus-group discussions were conducted between April and September 2018.

Table 2 provides the demographic characteristics for the 63 patient participants. One participant did not satisfy the terminal status of the 64 participants. The sample consisted primarily of older adults (59.3±8 years; range = 45–78 years) who were non-Hispanic (n = 58; 92.1%), White (n = 36; 57.1%), and female (n = 33; 52.4%). Over 41.0% (n = 26) of the sample was African American. About half the participants were married or living with a significant other (n = 31; 49.2%). The sample was fairly well educated with 39 (61.9%) reporting some college or university education or higher. Just over one half of the patient sample was employed full time (n = 24; 38.1%) or part time (n = 9; 14.3%) while 17 (27.0%) were retired and 10 (15.9%) were disabled.

**Table 2:** Patient demographic characteristics.

Patient Demographic Characteristics (Self-Reported)	Total (N = 63*)
**Age (years)**	
Mean (SD)	59.3 (8.0)
Median, range (min, max)	59 (45.0–78.0)
**Gender, n (%)**	
Male	29 (46.0%)
Female	33 (52.4%)
Missing	1 (1.6%)
**Ethnicity, n (%)**	
Not Hispanic or Latino	58 (92.1%)
Hispanic or Latino	2 (3.2%)
Missing	3 (4.8%)
**Racial background, n† (%)**	
American Indian or Alaska Native	1 (1.6%)
Asian	1 (1.6%)
Black or African American	26 (41.3%)
Native Hawaiian or other Pacific Islander	1 (1.6%)
White	36 (57.1%)
Other^^^	1 (1.6%)
**Relationship status, n (%)**	
Single	11 (17.5%)
Living alone	1 (1.6%)
Married	31 (49.2%)
Divorced/separated	13 (20.6%)
Widowed	5 (7.9%)
Other	2 (3.2%)
**Domestic living status, n (%)**	
Living in own home	62 (98.4%)
Living in an independent living community	0 (0.0%)
Living in an assisted living community	0 (0.0%)
Living in a nursing home or rehabilitation center	0 (0.0%)
Other	1 (1.6%)
**Employment status†, n (%)**	
Employed full-time	24 (38.1%)
Employed part-time	9 (14.3%)
Retired	17 (27.0%)
Disabled	10 (15.9%)
Homemaker	2 (3.2%)
Student	1 (1.6%)
Unemployed	2 (3.2%)
Other§	1 (1.6%)
**Highest levels of education†, n (%)**	
Some high school/no diploma	1 (1.6%)
Secondary/high school	9 (14.3%)
Some college/university	15 (23.8%)
Associate/vocational/technical degree	9 (14.3%)
College degree	24 (38.1%)
Postgraduate degree	4 (6.3%)
Other‖	3 (4.8%)

Abbreviation: SD = standard deviation.

* One patient did not satisfy terminal status.

† Responses are not mutually exclusive.

‡ Other race includes mix of African American and White.

§ Other employment includes temporary disability.

‖ Other education includes graduate school (n = 2).

Table 3 provides the clinical characteristics for the enrolled patients. Most patients were NYHA class II (n = 35; 55.6%) or III (n = 19; 30.2%). The mean duration since HF diagnosis was 8.9±8.5 years (range: 0.5 to 56.0 years). The most common self-reported comorbid conditions were hypertension (n = 34; 54.0%), arthritis (n = 28; 44.4%), myocardial infarction/heart attack (n = 14; 22.2%), anxiety (n = 12; 19.0%), depression (n = 10; 15.9%), and diabetes (n = 10; 15.9%).

**Table 3:** Patient clinical characteristics (self-reported).

Clinical Characteristics	Total (N = 63*)
**NYHA classification, n (%)**	
II	35 (55.6%)
III	19 (30.2%)
IV	8 (12.7%)
Missing	1 (1.6%)
**Duration since HF diagnosis (years)**	
Mean (SD)	8.9 (8.5)
Median (Range) (Interquartile range)	7 (0.5–56.0) (3–12)
Missing	1 (1.6%)
**Time since subject’s last hospitalization for HF (years)**	
Mean (SD)	3.6 (4.1)
Range	2 (0.0–16.6)
Missing	9 (14.3%)
**Duration of hospitalization (days)**	
Mean (SD)	4.4 (3.1)
Median (Range)	4 (0.0–14.0)
Missing	6 (9.5%)
**Comorbidities†**	
Anemia	8 (12.7%)
Angina	7 (11.1%)
Anxiety	12 (19.0%)
Arthritis	28 (44.4%)
Asthma	9 (14.3%)
Cancer	3 (4.8%)
COPD/emphysema	3 (4.8%)
Depression	10 (15.9%)
Diabetes mellitus	10 (15.9%)
Hypertension	34 (54.0%)
ICD only	10 (15.9%)
Myocardial infarction/heart attack	14 (22.2%)
PPM only	4 (6.3%)
Prior hemodialysis	0 (0.0%)
Stroke	4 (6.3%)
Transient ischemic attack	1 (1.6%)
None	2 (3.2%)
Other‡	13 (20.6%)
**Medications†**	
ACE inhibitors	22 (34.9%)
ARBs (or Inhibitors)	15 (23.8%)
Beta blockers	47 (74.6%)
Corlanor^®^	1 (1.6%)
Digitalis drugs	11 (17.5%)
Diuretics/water pills	31 (49.2%)
Entresto^®^	4 (6.3%)
Mineralocorticoid receptor antagonists	9 (14.3%)

Abbreviations: ACE = angiotensin-converting-enzyme; ARB = angiotensin II receptor blocker; COPD = chronic obstructive pulmonary disease; HF = heart failure; ICD = implantable cardiac defibrillator; NYHA = New York Heart Association; PPM = permanent pacemaker; SD = standard deviation.

*One patient did not satisfy terminal status.

† Not mutually exclusive.

‡ Other conditions include: Chronic inflammatory demyelinating polyneuropathy (Cidp), fibromyalgia, heart attack, leakage in two heart valves, PAD ischemic heart, Parkinson’s disease, atrial fibrillation, multiple sclerosis, myocardiopathy, sleep apnea, systemic fungal infection.

Table 4 provides a summary of the spontaneous and probed HF symptoms. All of the cardinal HF symptoms noted in past research were identified in this qualitative work. The most commonly-reported symptoms (reported by at least 20.0% of the patient sample) were shortness of breath (n = 52; 81.2%), fatigue/tiredness (n = 49; 76.6%), swelling of legs and ankles (n = 37; 57.8%), and trouble sleeping (n = 32; 50.0%).

*[Participant name 1*, *FG#1]: “the concomitant shortness of breath*, *that’s the #1 thing*. *It really plays havoc with a whole lot of other things*. *So the second one is the speed at which I get fatigued*. *And the length of time it takes to recover from being fatigued*. *And that is physical fatigue that–like [participant name 3] mentioned*, *it also really wipes out your ability to*, *like*, *stay focused*, *or even care to focus*.*”*

**Table 4:** Symptoms identified during patient qualitative focus groups (n = 64)*.

Symptoms†	N (%)
Shortness of breath (breathlessness/trouble breathing)	52 (81.2%)
Tiredness/fatigue	49 (76.6%)
Swelling of legs	37 (57.8%)
Trouble sleeping	32 (50.0%)
Chest pain	17 (26.6%)
Other bodily pain	13 (20.3%)
Cough	10 (15.6%)
Rapid heartbeat/palpitations	9 (14.1%)
Dizziness/lightheadedness	9 (14.1%)
Weight fluctuation	9 (14.1%)
Chest pressure/chest tightness	7 (10.9%)
General weakness	5 (7.8%)
Thinning hair	3 (4.7%)
Passing out/losing consciousness	3 (4.7%)
Sweating	3 (4.7%)
Stiffness	2 (3.1%)
Wheezing	2 (3.1%)
Lack of appetite/food no longer appealing	1 (1.6%)
Bloating	1 (1.6%)
Muscle tightness	1 (1.6%)
Muscle spasm	1 (1.6%)
Tingling sensation	1 (1.6%)

* Qualitative data from the patient who did not satisfy the terminal status was analyzed.

† Some of these self-reported symptoms could also reflect comorbid conditions (such as chest pain).

Table 5 provides a summary of the spontaneous and probed HF impacts from the patient perspective. A total of 47 day-to-day HF impacts were elicited from the patients. The top-three impacts were changes/reductions in social/family interactions (n = 43; 67.2%), dietary changes and restrictions (n = 41; 64.1%), and difficulty walking and climbing stairs (n = 36; 56.3%). Several mental-health sequelae were noted as day-to-day impacts including depression and sadness (n = 28; 43.8%), fear of dying (n = 21; 32.8%), anxiety (n = 21, 32.8%), and difficulty concentrating (n = 10; 15.7%). Other common physical impacts were: less exercise or low endurance for exercise (n = 31, 48.4%), being able to engage in recreational activities and hobbies (n = 28; 43.8%), difficulty performing work or job responsibilities (n = 17; 26.6%) or household chores (n = 15; 24.4%), difficulty lifting or carrying items (n = 13; 20.3%), needing frequent rests (n = 13; 20.3%), and frequent urination (n = 12; 18.8%). Eleven patients (17.2%) expressed that they disliked taking their medications predominantly due to side effects such as frequent urination.

*[participant name 1*, *FG#1]: “Life’s a chore a lot of the time*. *To go to the store is a chore*. *To go to dinner with friends is a chore*. *A lot of times*, *to be able to go out and fiddle around in my garden is a chore*. *To do the things that used to be able to give me joy are now chores*.*”*

*[participant name 2*, *FG#4]: “Well*, *I sure would like to exercise*. *I’m a big sports fan*, *but there’s just no way I can do what I used to do*, *I mean not even close*. *You know*, *that’s--that’s--that’s just very disheartening*. *I wouldn’t say depressing*, *but certainly it’s disheartening to see a lot of things I used to do and could do that*, *um*, *that I won’t even attempt to now*. *Because I know what the outcome will probably be*. *Um*, *socializing with friends and family*, *I kind of withdraw*, *because I know that when they’re ready to do certain things*, *I’m going to be no*, *I can’t really*, *you know*, *even come close to participating fully*. *So*, *uh*, *I take—I’d just rather not even get started*, *you know*, *and that’s kind of what I’m doing*. *Um*, *that’s*, *you know*, *pretty tough*. *Um*, *certain jobs I might want to do that I’m*, *uh*, *limited*, *that I know I won’t be able to do*, *you know*. *Um*, *and then*, *you know*, *my--the focus*. *Uh*, *a lot of times*, *um*, *you know*, *certain things I have to take care of*, *I need to--to keep really stay focused for a long time*, *really concentration level*. *Then*, *um*, *you know*, *once I feel those pains coming on*, *you know*, *that--that takes over everything*, *you know*. *That shoots right to the top of the list*, *and make sure I can get back to feeling normal*.*”*

*[004–107]: “Depression*. *Yeah*, *depression*, *that sets in real fast*, *because you can’t--you just feel that you’re worthless*, *that*, *you know*, *because of things*. *And you depend on people to do things for you*, *and I don’t like people doing things for me*. *It bugs me*. *But it’s just--it’s just depression*. *And everybody tries*. *Oh*, *the whole family tries to be nice and everything*, *and I wish they’d just go about being the way they were*. *You know*, *but it’s just that they just feel that you’re frail and you’re fragile and all*, *and it’s just*, *like they’ve been saying*, *people--people don’t understand*. *They--they just can’t comprehend it*.*”*

*[002–115]: “Um*, *just discomfort right after taking my medications*. *I don’t know what that’s all about*, *and it usually lasts for about 30 minutes*.*”*

**Table 5:** Impacts identified during patient qualitative focus groups (n = 64)*.

Impacts	N (%)
Social/family interactions	43 (67.2%)
Diet restrictions/changes	41 (64.1%)
Difficulty walking/inability to walk long distance/difficulty climbing stairs	36 (56.3%)
Difficulty exercising/low endurance	31 (48.4%)
Depressed/sadness	28 (43.8%)
Recreational activities/hobbies	28 (43.8%)
Scared/fear of dying	21 (32.8%)
Anxiety	21 (32.8%)
Forgetfulness/memory loss	20 (31.25%)
Performance of job/work	17 (26.6%)
Difficulty doing household chores/daily activities	15 (24.4%)
Ability to lift/carry items	13 (20.3%)
Needing frequent rests	13 (20.3%)
Frequent urination	12 (18.8%)
Stressed	11 (17.2%)
Dislike of medications	11 (17.2%)
Difficulty concentrating	10 (15.7%)
Getting around/mobility	10 (15.7%)
Isolation/alone	9 (14.1%)
Friends family pity	7 (11.1%)
Difficulty having sex/impact on sex life	7 (10.9%)
Coping (adapting/finding new ways to complete daily activities)	6 (9.4%)
Feeling like a burden	6 (9.4%)
Financial impacts	6 (9.4%)
Bending over	5 (7.8%)
Difficulty staying awake	5 (7.8%)
“Why me”	5 (7.8%)
Difficulty accepting diagnosis/denial/disbelief	5 (7.8%)
Frustrated/aggravated	5 (7.8%)
Mad/upset	5 (7.8%)
Embarrassment	5 (7.8%)
Frequent medical visits	5 (7.8%)
Defeated/hopeless	4 (6.3%)
Doubt condition will improve	4 (6.3%)
Lacking enthusiasm	4 (6.3%)
Disappointed	4 (6.3%)
Standing	4 (6.3%)
Lacking purpose	3 (4.7%)
Daunting/overwhelmed	3 (4.7%)
Difficulty with apparel and accessories	2 (3.1%)
Difficulty talking	1 (1.6%)
Difficulty laying down flat	1 (1.6%)

*Qualitative data from the patient who did not satisfy the terminal status was analyzed.

### Caregivers

Table 6 provides the demographic characteristics of 25 caregiver participants. A total of 26 caregivers were interviewed, but one participant did not fill out the sociodemographic form. Caregivers ranged in age from 33 to 71 years, with most identifying as non-Hispanic (n = 23, 92.0%), White (n = 19, 76.0%), and female (n = 13, 52.0%). The type of relationship to the HF patient included spouse/partner, child, sibling, or other relative; more than half (56.0%) of the caregivers lived with the HF patient. Caregivers were well educated with almost all (96.0%) reporting some college or higher.

**Table 6:** Caregiver demographic characteristics.

Caregiver Demographic Characteristics (Self-Reported)	Total (N = 25*)
**Age (years) of caregiver**	
Mean (SD)	55.5 (11.2)
Median, range (min, max)	59 (33.0–71.0)
Missing	1 (4.0%)
**Age (years) of HF patient cared for by caregiver**	
Mean (SD)	73.8 (10.3)
Median, range (min, max)	73 (52.0–90.0)
Missing	1 (4.0%)
**Gender, n (%)**	
Male	11 (44.0%)
Female	13 (52.0%)
Missing	1 (4.0%)
**Ethnicity, n (%)**	
Not Hispanic or Latino	23 (92.0%)
Hispanic or Latino	0 (0.0%)
Missing	2 (8.0%)
**Racial background†, n (%)**	
American Indian or Alaska Native	1 (4.0%)
Asian	1 (4.0%)
Black or African American	4 (16.0%)
Native Hawaiian or other Pacific Islander	1 (4.0%)
White	19 (76.0%)
Other‡	1 (4.0%)
**Relationship with the HF patient, n (%)**	
Spouse/partner	8 (32.0%)
Sibling	3 (12.0%)
Child	6 (24.0%)
Other relative	3 (12.0%)
Friend or non-related caregiver	2 (8.0%)
Other§	1 (4.0%)
Missing	2 (8.0%)
**Living with HF patient, n (%)**	
Yes	14 (56.0%)
No	10 (40.0%)
Missing	1 (40.0%)
**Domestic living status, n (%)**	
Living in own home	24 (96.0%)
Living in an independent living community	0 (0.0%)
Living in an assisted living community	0 (0.0%)
Living in a nursing home or rehabilitation center	0 (0.0%)
Missing	1 (4.0%)
**Employment status, n (%)**	
Employed full-time	14 (56.0%)
Employed part-time	7 (28.0%)
Retired	3 (12.0%)
Disabled	0 (0.0%)
Homemaker	0 (0.0%)
Student	0 (0.0%)
Unemployed	0 (0.0%)
Other	0 (0.0%)
Missing	1 (4.0%)
**Highest levels of education, n (%)**	
Some high school/no diploma	0 (0.0%)
Secondary/high school	0 (0.0%)
Some college/university	6 (24.0%)
Associate/vocational/technical degree	3 (12.0%)
College degree	14 (56.0%)
Postgraduate degree	2 (8.0%)
Other	0 (0.0%)
Missing	1 (4.0%)

Abbreviations: HF = heart failure; SD = standard deviation.

* Not mutually exclusive.

† One participant did not complete the sociodemographic form.

‡ Other race: Bi-racial.

§ Other relationship to patient: my mother.

Table 7 provides a summary of the spontaneous and probed HF impacts and consequences from the caregiver perspective. A total of 33 day-to-day HF impacts were elicited from caregivers. The top impacts (greater than 20% endorsement) were changes/reductions in social/family interactions (n = 13; 50.0%); being stressed, worried, and fearful (n = 12; 46.2%) and having to monitor their “patience” level (n = 11; 42.3%). Eleven caregivers (42.3%) mentioned that they used vacation time for caregiving, made less money if they called off work, transitioned to part-time work, or retired early to meet the responsibilities of caring for their patient. Caregivers often expressed that they had difficulty performing work or job responsibilities or regular daily activities (n = 11; 42.3% negative/suboptimal changes in recreational activities and hobbies (n = 10; 38.5%); being frustrated (n = 9; 34.6%); physical consequences of being tired or exhausted (n = 8; 30.8%); sacrificing sleep, having insomnia, or trouble sleeping (n = 8; 30.8%); depression (n = 7; 26.9%); and feeling one is being taken for granted or feeling unappreciated (n = 6; 24.0%). Two caregivers (7.7%) expressed that their caregivee’s had “slowed down” because of HF, often needing frequent rests and not being as agile as they used to be with respect to their physical abilities. Three caregivers (18.8%) expressed that their patient often fell off the furniture because of weakness they experience.

*[003–202]: “Because he used to walk around the house with no issues*. *Now he is a little bit slow and he is feeling vulnerable in case he is at home at the house by himself*, *and he may have a fall and*, *uh*, *things like that*.*”*

**Table 7:** Consequences on caregivers daily lives.

Symptom Consequences	Caregivers (N = 26)
**Social Consequences**	
Social/family interactions	13 (50.0%)
Recreational activities/hobbies	10 (38.5%)
Closer relationship to patient	2 (7.7%)
Social isolation	2 (7.7%)
**Emotional Consequences (Positive and Negative)**	
Stressed/worried/fearful	12 (46.2%)
Patience	11 (42.3%)
Frustrated	9 (34.6%)
Depression	7 (26.9%)
Lack of appreciation/taken for granted^1^	6 (24.0%)
Hypervigilance	5 (19.2%)
Exhausting/mentally draining	3 (18.75%)
Resentful	3 (18.75%)
Empathy/sympathy^1^	3 (18.75%)
Guilt	4 (15.4%)
Lack of freedom	4 (15.4%)
Staying in the moment, nagging, “hardened,” crabby, more in routine, or facing one’s own mortality	2 (7.7%)
Religious, not as happy go luck, forgiving, confused, or encouragement	1 (3.8%)
**Financial Consequences**	
Performance of daily activities/job/work	11 (42.3%)
Adequate insurance coverage (Positive)	5 (19.2%)
Medication co-pays*	2 (7.7%)
Hiring aide	2 (7.7%)
Transportation/gas	1 (3.8%)
Grocery food shopping	1 (3.8%)
Costs associated with having another person in the house*	1 (3.8%)
**Physical Consequences**	
Tired/exhausted	8 (30.8%)
Sacrifice sleep/insomnia/trouble sleeping	8 (30.8%)
Weight fluctuations	2 (7.7%)
Unhealthy diet	2 (7.7%)
Exercise more	1 (3.8%)
Missed healthcare visits	1 (3.8%)
Better diet	1 (3.8%)

* Number of participants who endorsed topic includes quotes from overlapping conversations where specific IDs were difficult to parse out.

Below are some illustrative quotes that reflect caregiver impacts.

*[003–04 –participant name 1] Um*, *I become more busy*. *It’s like having a child*, *pretty much*. *I have to look at him all the time*, *look after him all the time*. *So especially at night when he is at sleep*, *because he can’t go to the bathroom*, *so I have to check on him in the middle of the night to make sure that has been taken care of*.

*[003–04 –participant name 4] The physical challenge is definitely*, *you know*, *because my parents live 110 miles away from me*. *So*, *you know*, *I get five calls a day about stuff that’s–in the scope of things*, *is pretty insignificant*, *but for them it’s like major*, *so I’ve had to literally–and I work in sales*. *I have to drop my career to tend to them*, *you know*, *on a moment’s notice*. *That’s really frustrating*. *…It’s a challenge because*, *you know*, *I have a family myself…– it’s almost like putting your finger in a dam*, *you know*, *just try to cover as many holes as you can and hope it doesn’t flood*.

*[002–211] Tired*, *crabby*, *aggravated*, *giving up your social life on weekends*.

*[002–207] Everywhere I turn it’s like another problem*.

*[002–208] He doesn’t like me having ‘me’ time*. *I like to read*. *I’ll pull out a book*, *and he’s irritated*. *Why are you not talking to me? Why do you want to always read? Well*, *because you’re boring*.

*[002–208] So it’s like my freedom is—is gone*. *So I’m always washing his clothes [due to urination accidents]*, *which is a pain*. *Um*, *I—I retired and I was hoping to do some traveling*. *So I guess I’m sad because I thought my retirement would be different*, *um*, *so as a result*, *I’m not having time for myself to do my walking*, *bicycle riding*. *In the summer I like to go bicycle riding, and then he gets sad because I’m leaving him and I’m going on my bike*. *I have little time for myself and my interests*, *I’m becoming more like a boring person*, *myself*, *um*, *um*, *and I used to be really*, *uh*, *outgoing and*, *um*, *kind of free spirit*. *Well*, *that’s not so much anymore*, *so I think my personality has changed*.

*002-207e: Well*, *the—the problem would be I’d have to leave the house*, *and then he’d want to know why aren’t you here with me*, *where are you going?*

*002–201: It’s very discouraging*. *Um*, *I’m always on edge*. *It’s hard to sleep*. *You’re always wondering*, *you know*, *if something’s going to happen*. *You know*, *you’re--it’s just*, *um*, *very*, *very*, *um*, *mentally and physically draining*.

*002–204: I’m a pretty positive person*. *I’m always laughing and smiling*. *It’s always an act*. *It’s always an act*, *because if I show people that I’m sad and depressed*, *it’s not going to help me*. *And it’s not going to help my husband*.

*002–206: I can’t make any plans*, *can’t plan ahead*. *Um*, *can’t leave him unattended*, *can’t leave him alone*. *That’s a given*. *Um*, *and even when I do*, *it’s [laughter] why aren’t you answering your phone*, *um*, *you know*. *You know*, *it’s--I put exhausting*. *It’s overwhelming…no time for myself*.

*002–203: The constant worrying*, *because almost every time he goes to the doctor it’s a new problem or something getting worse*. *Um*, *feeling tired constantly*, *um*, *loss of friends and no time for myself*, *and kind of a thankless job*.

*002–201: Well*, *taken for granted*. *God*, *you know*, *you’re here but nobody really appreciates it*, *because they--and then*, *of course*, *you know*, *they take out their anger and frustration on you*. *Um*, *so it’s very stressful*, *can’t make plans*.

*004–205: It’s the time*. *I cannot leave the house for any length of time without getting 100 phones calls*. *Um*, *the financial cost of having someone come in so that I can get a few hours*, *that costs*. *Um*, *he also has*--*my father also has constant fears*. *And the complaints*, *he just--it--it causes a tremendous amount of stress*, *which translates into my job*, *and then I have to call out*. *It’s a lot*.

*004–207: I’m an anxious mess*. *Um*, *I’ve lost weight*. *I don’t sleep well anymore*. *Um*, *definitely get bouts of depression*, *and*, *um*, *anxiety*

## Discussion

Living with and managing HF is a “shared experience” [14] and is demanding and arduous “work” [16, 24] for patients and their caregivers. The HF journey adversely impacts patient and caregiver physical, mental, and social well-being and can bring about fear, uncertainty, depression, anxiety, and isolation. Understanding and addressing the totality of these experiences is a first step to improving patients’ and caregivers’ symptom ramifications.

This qualitative study confirmed previous research about the cardinal HF symptoms from the patient perspective and their daily impacts on patients with HF. These HF cardinal symptoms (from the patient perspective) were shortness of breath, tiredness and fatigue, edema, and difficulty sleeping [4, 26–37]. Because the physical symptoms of HF can be so debilitating and incapacitating, several patient-preference studies have shown that HF patients value symptom stabilization or improvement (especially with respect to dyspnea, fatigue, and physical functioning) over outcomes such as hospitalization and increased risk of mortality [8, 9, 38–40]. In a qualitative, focus group study, Kraai and colleagues [41] reported that decreased symptoms, physical functioning, prevention of hospital readmissions, and living a normal life were the HF treatment goals most important to patients; none of the participants mentioned improved survival as a treatment goal. This research also corroborated the mental-health sequelae associated with HF reported by others including depression [5, 7, 29, 32, 34, 42, 43] and anxiety [29, 33, 34]. Patients often struggle with their new identity as a person with HF as well as its barriers on daily and normal activities that were once done without forethought and planning.

The findings of the current qualitative study further support the results of other studies demonstrating that the frequency and severity of HF-related symptoms (i.e., shortness of breath, fatigue, edema, etc.) impact physical, emotional and social functioning and well-being and results in significantly impaired HRQoL [4, 28, 44, 45]. This qualitative research with 64 patients and 26 caregivers provides contemporary information on these impacts based on the perspective of the patients themselves and helps to provide a more real-life interpretation of these impacts of symptoms on functioning and well-being.

This qualitative research poignantly described the lived experiences of being a caregiver of patients with HF. Caregivers play a key and meaningful role in their caregivee’s HF self-care and HF health outcomes [20]. However, most of the caregivers interviewed in this study often felt they were on their own, left to their own devices, and lacked support and training for their new roles. Many of the caregivers interviewed in this study had to make significant changes to their daily life and routine including early retirement or reduced employment hours. Many also reported a significant amount of stress, and often social and emotional isolation, associated with caregiving for a patient with HF. Caregivers often had to assume additional daily roles and responsibilities due to their caregivee’s functional and psychosocial limitations. Many caregivers experienced caregiver “overload” and experienced their own recurring problems with fragile emotional and physical health, excessive stress, and problems with sleep. Intermittent periods of resentment and feeling unappreciated/taken for granted (e.g., being a forced volunteer [13] and an unsung hero [17]) were common among the caregivers studied herein.

This study provides evidence of patient and caregiver unmet needs in HF which are poorly understood by clinicians and vastly underserved by the healthcare system. These unmet needs require interventions that not only meet the diverse cultural needs of patients and their caregivers but also incorporates individual preferences for optimizing health outcomes that mean the most to patients and caregivers.

### Limitations

This qualitative study has several limitations. First, the mean duration of living with HF was 8.9 years. By the virtue of surviving this time, these patients may have generally had the opportunity to find a new equilibrium with their disease and its impact on their caregivers. Thus, these findings may not be extended to patients living with HF for fewer years. Next, small sample sizes—which can limit generalizability and external validity—are often a characteristic of qualitative research as was the case in this study. However, our sample of 64 patients and 26 caregivers was considerably larger than that of past research. The sample of interviewed patients and caregivers was fairly well educated, relatively young, underrepresented participants from Hispanic origin and resided in metropolitan cities It is unknown whether the burden and impacts reported herein would have been exacerbated or attenuated among more vulnerable HF patients and their caregivers. The key phenotypes of heart failure (i.e., HF with preserved vs. reduced ejection fraction) were not distinguished in sampling. Our sample of patients and caregivers were not paired or matched as was the case with past research. No qualitative subgroup analyses were conducted (e.g., by gender or age). Finally, 56.0% of the patients self-reported NYHA class II, 30.0% self-reported class III, and 13.0% self-reported class IV. It is unknown if the symptoms and impacts revealed herein would have been intensified if more symptomatic patients and their caregivers were sampled.

## Conclusions

In conclusion, living with HF is a shared—and often demanding—journey between patients and their caregivers. Patients experience many distressing and burdensome HF symptoms and feel they have a detrimental and deleterious impact on their daily functioning and well-being. Many caregivers are overloaded and stressed and suffer from harmful, negative impacts on their physical and emotional health. The direct costs of HF only take into consideration objective metrics such as healthcare utilization and expenditures. Even measures of indirect costs, while slightly more patient- and caregiver-centered, do not account for or incorporate the distressing and disruptive deficits in functioning and well-being that patients and caregivers experience. As shown by this and other research, the magnitude of patient and caregiver unmet need is palpable. More systematic research is needed to better characterize and understand unmet need at the patient, caregiver, and societal level. We advocate for making HF a much higher priority—perhaps akin to the oncology patient-centered medical homes—with quality metrics and policy changes that can help ameliorate the daily suffering and broad societal impact that is currently vastly underestimated and undertreated.

## Supporting information

S1 Appendix (DOCX)
