# Supplementary figures and images for: The impact of heart failure on patients and caregivers: A qualitative study
Source: PLoS One. 2021 Mar 11;16(3):e0248240. doi: 10.1371/journal.pone.0248240 (PMC7951849; doi:10.1371/journal.pone.0248240)

# S1 Appendix: Posters used with the focus group discussion guide


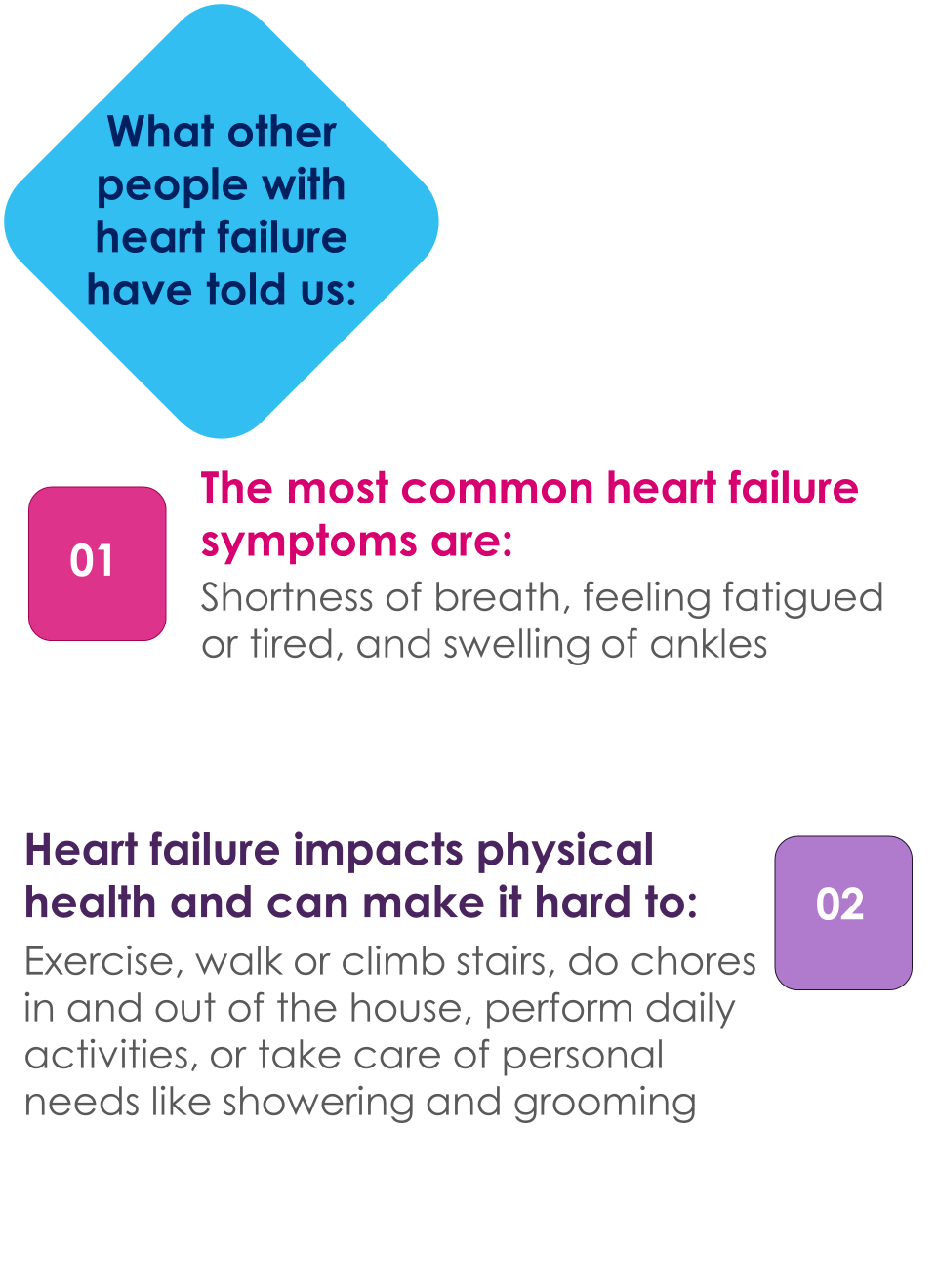


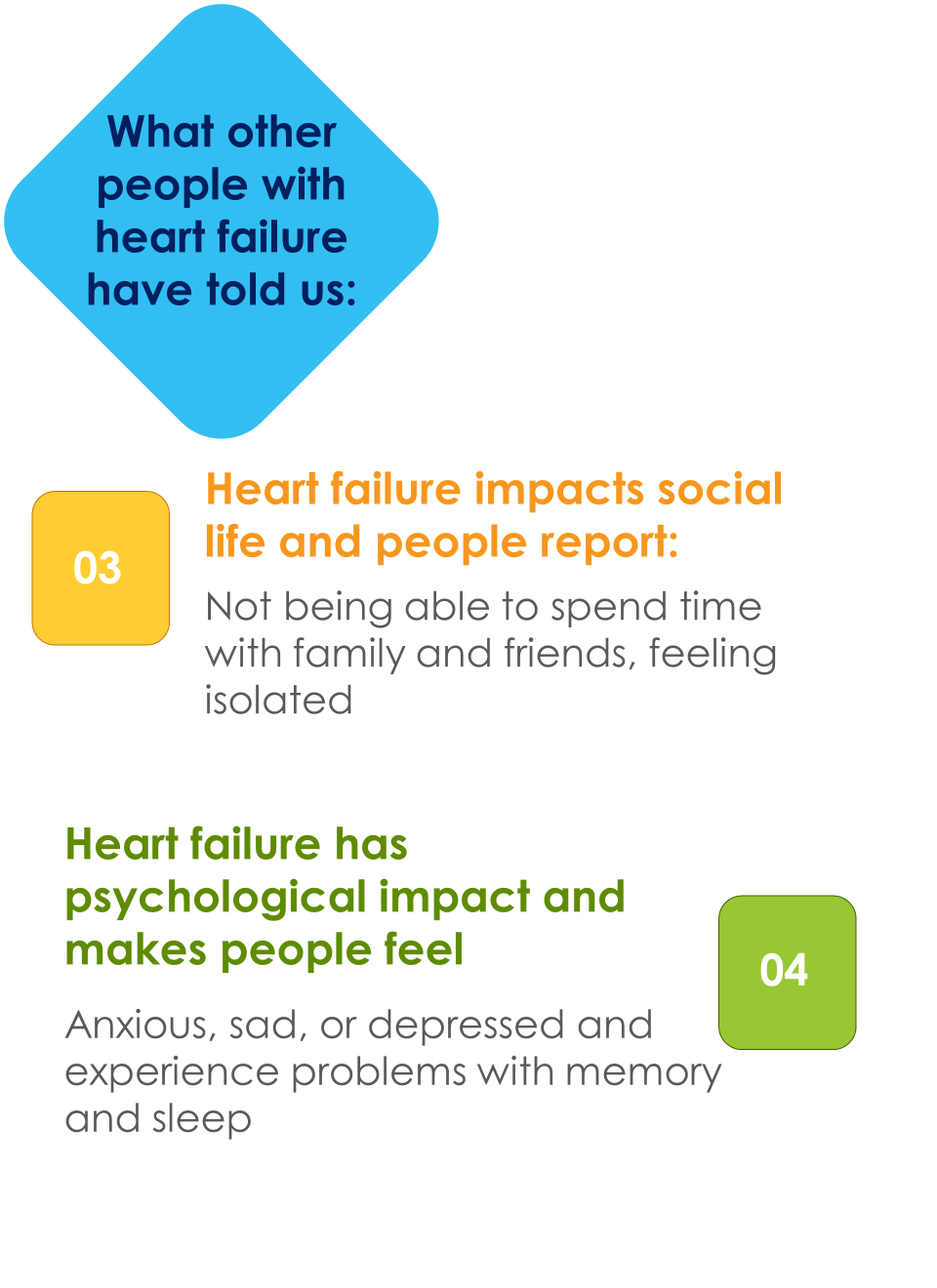

Supplement: S1 Appendix — (DOCX) [file pone.0248240.s001.docx]
